# EFFECTS OF CIVIC BEHAVIOR AND DEPRESSION KNOWLEDGE ON DEPRESSION STIGMA AMONG OLDER CHINESE: A PATH ANALYSIS MODEL

**DOI:** 10.1093/geroni/igad104.2869

**Published:** 2023-12-21

**Authors:** Jessie Ho-Yin Yau, Tianyin Liu, Dara Kiu Yi Leung, Jennifer Yee-man Tang, Gloria Hoi-Yan Wong, Terry Lum

**Affiliations:** The University of Hong Kong, Hong Kong, Hong Kong; The University of Hong Kong, Hong Kong, Hong Kong; The University of Hong Kong, Hong Kong, Hong Kong; The University of Hong Kong, Hong Kong, Hong Kong; The University of Hong Kong, Hong Kong, Hong Kong; The University of Hong Kong, Hong Kong, Hong Kong

## Abstract

Older adults are at risk of experiencing depression personal stigma because of negative attitudes towards older persons and the creation of popular stereotypes, therefore reducing stigma becomes necessary. By receiving training and acquiring depression knowledge from engaging in civic behavior, such as providing voluntary services to peers in the community, these knowledge helps counter false assumptions and reduce personal stigma. However, previous stigma-related studies mainly conducted in Western countries and in relation to schizophrenia, research on depression stigma among older Chinese remain scarce. The goal of this study was to examine depression knowledge as a moderator to test its influence on the relationship between civic behavior and personal stigma. A cross sectional survey was conducted with 145 older adults in Hong Kong, they completed Older Adult Mental Health First Aid training and engaged in voluntary services related to mental health promotion after the training. Civic behavior, depression knowledge (symptoms, facts, myths), personal stigma (stereotype, prejudice, discrimination), and demographics were collected. Path analysis was performed to determine the structural relationship between the variables. Civic behavior positively affected symptoms (β=.089,p=.008) and facts (β=.088,p=.001). Symptoms negatively affected prejudice (β=-.627,p=.014). Facts negatively affected all dimensions of personal stigma while myths positively affected all dimensions of personal stigma. Results of the measurement showed an excellent fit (χ2 = 148, χ2 /df = 7.048, RMSEA = .029, SRMR = .023, CFI = 0.997, TLI = 0.98, p<.001). Mediating effects were computed by bootstrap method; facts as a significant mediation was confirmed. Findings and implications were discussed.

